# Spatial Assessment of Amphibian Chytrid Fungus (*Batrachochytrium dendrobatidis*) in South Africa Confirms Endemic and Widespread Infection

**DOI:** 10.1371/journal.pone.0069591

**Published:** 2013-07-22

**Authors:** Jeanne Tarrant, Dirk Cilliers, Louis H. du Preez, Ché Weldon

**Affiliations:** Unit for Environmental Sciences and Management, North-West University, Potchefstroom, North West Province, South Africa; University of Kent, United Kingdom

## Abstract

Chytridiomycosis has been identified as a major cause of global amphibian declines. Despite widespread evidence of *Batrachochytrium dendrobatidis* infection in South African frogs, sampling for this disease has not focused on threatened species, or whether this pathogen poses a disease risk to these species. This study assessed the occurrence of *Bd*-infection in South African Red List species. In addition, all known records of infection from South Africa were used to model the ecological niche of *Bd* to provide a better understanding of spatial patterns and associated disease risk. Presence and prevalence of *Bd* was determined through quantitative real-time PCR of 360 skin swab samples from 17 threatened species from 38 sites across the country. Average prevalence was 14.8% for threatened species, with pathogen load varying considerably between species. MaxEnt was used to model the predicted distribution of *Bd* based on 683 positive records for South Africa. The resultant probability threshold map indicated that *Bd* is largely restricted to the wet eastern and coastal regions of South Africa. A lack of observed adverse impacts on wild threatened populations supports the endemic pathogen hypothesis for southern Africa. However, all threatened species occur within the limits of the predicted distribution for *Bd*, exposing them to potential *Bd*-associated risk factors. Predicting pathogen distribution patterns and potential impact is increasingly important for prioritising research and guiding management decisions.

## Introduction

Large-scale, enigmatic amphibian declines in the 1990 s in undisturbed regions of the tropics resulted in the identification and description of a novel chytrid fungus *Batrachochytrium dendrobatidis* (hereafter *Bd*) [1]. This non-hyphal zoosporic fungus causes the skin disease, chytridiomycosis, in amphibians and is now recognised as a significant contributor to global amphibian declines [2], [3], [4], [5]. The pathogen has to date been detected in over 500 amphibian species worldwide [6], approximately half of which are species experiencing population declines [3], [7], [8] and has also now been linked to several species extinctions [8], [9], [10]. Strategies to protect amphibians from this disease are therefore essential in the overall campaign to conserve amphibians.

As of the most recent IUCN Red List assessment, 32.4% of 6260 amphibian species are globally threatened (in the categories Vulnerable, Endangered, or Critically Endangered) or Extinct [11]. These trends are reflected in South Africa, with 29% of frog species listed as threatened [12], [13]. These threatened taxa are distributed through five provinces in South Africa, namely Western Cape (eight species), KwaZulu-Natal (six species), Eastern Cape (three species), Northern Cape (one species) and Limpopo (one species). While loss of habitat due to land transformation has been identified as the most pervasive threat to these species [12], [14], [15], the potential threat of *Bd* infection in threatened South African frogs has to date not been investigated.

The presence of endemic *Bd* in Africa in the 1930 s, together with *Xenopus* spp. exports provided evidence for a pathogen emergence hypothesis [16], [17], [18]. Subsequent detection of *Bd* with no adverse effects in wild populations from central and east Africa support this hypothesis [19], [20], [21], [22], . However, failure to detect *Bd* in West Africa and *Bd*-related population declines in one species from East Africa [24], [25] challenge this hypothesis. Despite the long-term presence of *Bd* in South Africa little has been done by way of investigating the threat it may pose to indigenous species, in particular those that are Red Listed. No consideration was given to the possible threat of infectious disease to any of the species that were reviewed during the last Red List Assessment of South African frogs [26].

Understanding the distribution of *Bd* within a region is paramount for implementing protocols for disease management and for determining disease or extinction risk [27]. Ecological niche modelling has become an increasingly used tool for predicting *Bd* distribution and associated disease risk on both a global [28] and regional scale [29], [30], [31].

Because *Bd’s* ecological preferences are well understood [32], [33], the use of ecological niche modelling provides an ideal tool for predicting potential distribution of this pathogen. Furthermore, unlike many other pathogens which rely on specific internal host conditions, *Bd* is an ideal candidate for modelling since infections occur on ecothermic amphibian hosts and the pathogen is directly influenced by external environmental conditions, especially temperature and moisture [34].

Former spatial assessments of *Bd* [e.g. 28, 29] have limited application in South Africa, because they either do not include records from South Africa or were based on a small number of samples with a limited range, resulting in too broad a spatial scale to be useful on a regional scale. Therefore predictions on a finer scale, using up-to-date data, are necessary to assess *Bd* distribution in South Africa. This study makes use of a comprehensive database of *Bd infection* of frogs to predict where the fungus is likely to occur in amphibian hosts within the country. The aims of this study were to assess the occurrence of *Bd*-infection in South Africa’s threatened frogs, and to model the predicted distribution of *Bd* to improve the understanding of which species and regions may be at higher infection risk.

## Materials and Methods

### Prevalence Assessment

Swab samples from threatened South African frog species (IUCN Red List categories: Vulnerable, Endangered and Critically Endangered) were collected at known historic sites known from the South African frog atlas [25] as well as from new sites where the target species occur, which were detected during the course of the field work between 2008 and 2012, primarily during the rainy months (August – March). Individuals of each species were detected via visual, acoustic or opportunistic searches and caught by hand or net. Sample size per site depended on detection likelihood of the target species, but where possible, 20 samples of each species were taken per site (Table 1). This sample size is based on an assumption about the likely prevalence within the sampled population, which is then factored into the equation given a specific confidence interval (e.g. 95%). The result is a minimum sample size that should be screened in order to say with 95% confidence that *Bd* is not present in the sample when all samples test negative. Thus the lower the assumed prevalence before sampling, the higher the required sample size becomes and vice versa. There generally is high prevalence in populations where *Bd* is present in South Africa and therefore the target of 20 animals. A second reason for this relatively small sample target was because we were working with threatened species that often occur in low densities, and 20 individuals is a realistic target given the likelihood of finding the animals.

**Table 1:** Locality data for *Batrachochytrium dendrobatidis* testing in threatened South African frog species, by province.

Site Name	Province	Latitude	Longitude	Target species	Red List Category 2010	*N*
Coffee Bay	EC	−31.93496	29.08826	*Afrixalus spinifrons*	NT	1
Dwesa-Cwebe NR	EC	−32.25348	28.87046	*Natalobatrachus bonebergi*	EN	1
Geelhoutboom River	EC	−33.79434	25.06377	*Heleophryne hewitti*	EN	32
Hogsback A	EC	−32.59892	26.94552	*Anhydrophryne rattrayi*	EN	3
Hogsback B	EC	−32.54774	26.91443	*Vandijkophrynus amatolicus*	CR	1
Martins River	EC	−33.79326	25.03819	*Heleophryne hewitti*	EN	2
Adam's Mission	KZN	−29.99183	30.78328	*Hyperolius pickersgilli*	CR	1
Cato River	KZN			*Hemisus guttatus*	VU	2
Cedara	KZN	−29.55784	30.255406	*Afrixalus spinifrons*	NT	1
Cowies Hill	KZN	−29.82436	30.59567	*Natalobatrachus bonebergi*	EN	1
Mtunzini	KZN	−28.96782	31.75322	*Hyperolius pickersgilli*	CR	4
Fort Nottingham	KZN	−29.4449	29.90642	*Afrixalus spinifrons*	NT	9
Hilton	KZN	−29.53916	30.28625	*Afrixalus spinifrons*	NT	2
Isipingo	KZN	−29.99185	30.9056	*Hyperolius pickersgilli*	CR	46
Kamberg NR	KZN	−29.37361	29.725	*Afrixalus spinifrons*	NT	3
Lake Merthley	KZN	−29.02242	30.58106	*Leptopelis xenodactylus*	EN	10
Mt. Moreland	KZN	−29.6382	31.09754	*Hyperolius pickersgilli*	CR	28
Port Durnford	KZN	−28.90521	31.85801	*Hyperolius pickersgilli*	CR	6
Prospecton	KZN	−29.98328	30.938	*Hyperolius pickersgilli*	CR	14
Rosetta	KZN	−29.30417	29.9625	*Afrixalus spinifrons*	NT	2
Tala NR	KZN	−29.82954	30.53535	*Afrixalus spinifrons*	NT	2
Umlalazi NR	KZN	−28.95805	31.76472	*Hyperolius pickersgilli*	CR	1
Vernon Crookes NR	KZN	−30.2786	30.59596	*Natalobatrachus bonebergi*	EN	17
Widenham	KZN	−30.21718	30.795353	*Hyperolius pickersgilli*	CR	1
Haernertsburg	LP	−23.93619	29.93916	*Breviceps sylvestris*	EN	2
Hanglip	LP	−22.99959	29.88359	*Breviceps sylvestris*	EN	1
Soutspanberg	LP	−22.99599	29.88353	*Breviceps sylvestris*	EN	2
Woodbush	LP	−23.81111	29.96365	*Breviceps sylvestris*	EN	16
MacDougal's Bay	NC	−29.26172	16.87107	*Breviceps macrops*	VU	4
Bergvliet	WC	−34.04864	18.44789	*Amietophrynus pantherinus*	EN	22
Cape Agulhas	WC	−34.74106	19.67883	*Xenopus gilli*	EN	25
Cape Point	WC	−34.30603	18.44133	*Xenopus gilli*	EN	25
Disa Stream	WC	−33.98586	18.39072	*Heleophryne rosei*	CR	26
Kennilworth	WC	−33.99637	18.48486	*Microbatrachella capensis*	CR	20
Kirstenhof	WC	−34.08555	18.4525	*Amietophrynus pantherinus*	EN	7
Noordhoek	WC			*Hyperolius horstocki*	VU	5
Silvermine NR	WC	−34.10095	18.44809	*Capensibufo rosei*	VU	35
Skeleton Gorge	WC	−33.98586	18.39072	*Heleophryne rosei*	CR	8
University of Cape Town	WC	−33.95818	18.45746	*Breviceps gibbosus*	NT	2
Youngsfield Military Base	WC	−34.00419	18.49025	*Amietophrynus pantherinus*	EN	2
**40 Sites**	**5 Provinces**			**17 Species**		**392 samples**

EC = Eastern Cape; KZN = KwaZulu-Natal; LP = Limpopo Province; NC = Northern Cape; WC = Western Cape.

NR = Nature Reserve.

Field sampling protocol follows [35] (but swabs were air-dried and not placed into alcohol). A fresh pair of latex gloves was used for each animal swabbed. The cotton tip of the swab was gently stroked five times each over the ventral surfaces of the thighs, tibia, ventrum and webbing of the frog, which was then released at the point of capture. Re-sampling of the same individual was avoided by keeping individuals in separate bags and postponing swabbing until all frogs had been captured. Swabs were kept refrigerated at approximately 4°C until testing. Equipment and footwear was cleaned and disinfected with 5% bleach solution at the commencement of fieldwork and between sites by following the hygiene protocol of [36].

### Molecular Diagnosis of Batrachochytrium Dendrobatidis

Real-time TaqMan PCR was used to detect *Bd* according to the international standard protocol [8]. DNA from skin swabs was extracted using PrepMan Ultra (Applied Biosystems™, Foster City, CA) and analysed for *Bd* using quantitative real-time TaqMan PCR assays [37] with *Bd-*specific primers *Bd*1a (5′-CAGTGTGCCATATGTCACG-3′) and *Bd*2a (5′-CATGGTTCATATCTGTCCAG-3′) [38]. The StepOnePlus™ real-time PCR system from Applied Biosystems™ was used for the TaqMan assay. Samples were processed in duplicate and standards for quantification of *Bd* followed [37]. Amplification in both reactions was considered positive, while no amplification was considered negative.

### Predictive Distribution Modelling

Predictive modelling software MaxEnt ver. 3.3.3k [39] was used to model the predicted distribution of *Bd* in South Africa. Maxent output correlates environmental suitability for the target organism, where higher values correspond to a better prediction of better conditions [40]. MaxEnt combines presence-only data [41], [42] with spatially explicit environmental variables [43] to predict species distribution for a given study area. Several studies [39], [42], [44], [45], [46] have proven the effectiveness of MaxEnt as a distribution modelling approach [47], [48] and confirmed its usability for such purposes. See [38] statistical and technical discussion on MaxEnt. One hundred and twenty six (excluding duplicates) *Bd-*positive presence records with high resolution geo-referencing data (GPS co-ordinates) were compiled from the Africa *Bd* database (unpublished data), including all samples from this study.

The environmental variables used for the MaxEnt model were bioclimatic variables obtained from BIOCLIM [49], topographic variables derived from an SRTM digital elevation model (DEM) and biome data obtained from the South African National Biodiversity Institute [50]. For the initial modelling, 24 variables were used, of which 19 were bioclimatic variables (continuous), four geo-physical variables and one a biome-type variable (categorical). Of these, 12 variables with the highest contribution to the model were selected for the final run (Table 2). The variables were re-sampled to 250 m grids using ArcGIS v10 [51]. The following model parameters were used: to allow adequate time for convergence, the number of iterations were set to 5000; number of replicates was set to 100 using bootstrapping with 30% of the data used for testing and 70% for training. In an effort to reduce sampling bias, a bias file was used to guide background point selection [52]. Model performance was evaluated by the area under the curve (AUC) statistic of the receiver operating characteristic (ROC) plots [39], [53]. Jack-knife tests of variable importance, using regularised training gain, test gain and AUC for test data, determined which variables made the greatest contribution to the model. From the results of the first model the top ranking variables contributing 90% of the information for the model were identified and used in subsequent models. Four further models were run using the above parameters, but with different combinations of environmental variables. Redundant environmental variables were excluded based on the Jack-knife analysis results of previous models.

**Table 2:** Environmental variables, and their percentage contribution, included in the final MaxEnt niche model for predicted distribution of *Batrachochytrium dendrobatidis* in South Africa.

Key	Variable	Contribution to final model (%)
BIO2	Mean Diurnal Range (Mean of monthly (max temp – min temp)	5.3
BIO9	Mean Temperature of Driest Quarter (°C)	6.4
BIO10	Mean Temperature of Warmest Quarter (°C)	3.7
BIO11	Mean Temperature of Coldest Quarter (°C)	11.8
BIO15	Precipitation Seasonality (Coefficient of Variation)	5.2
BIO16	Precipitation of Wettest Quarter (mm)	8.5
BIO19	Precipitation of Coldest Quarter (mm)	9
Topo	Topography	19.6
dist to water	Distance to water	16.8
Biomes	Biomes	5.1
Slope	Slope	3
Aspect	Aspect	5.5

### Ethics

Swab sampling provides a non-invasive method of testing for *Bd* whereby frogs are not harmed in any way and released shortly after sampling. Ethics clearance for related research was provided by RESEARCH ETHICS APPLICATION - NWU-00013-10-S4, North-West University. Research permits were provided by Ezemvelo KZN Wildlife (Permit Nos. 4485/2008, 4137/2008, OP 64/2010, OP 1180/2010 and 5080/2011); iSimangaliso Wetland Park Authority; SANParks (Table Mountain National Park, Agulhas National Park, Namaqua National Park), Cape Nature for the Western Cape Province (Permit No. AAA006-00022-0035 and AAA-004-00046+47-0035) and Eastern Cape Parks & Tourism Agency (Permit No. RA 0109). Permit 028 NW-11 of the Department: Economic Development, Environment, Conservation and Tourism, North West Provincial Government. Permit WRO 37/03WR of the Department of Ecolonomic Affairs, Environment and Tourism, Province of the Eastern Cape. Permit 001-CPM403-00004 issued by Department of Economic Development, Environment And Tourism, Limpopo Provincial Government.

## Results

### Prevalence of Batrachochytrium Dendrobatidis Infection in South Africa’s Threatened Frogs

**Figure 1:**
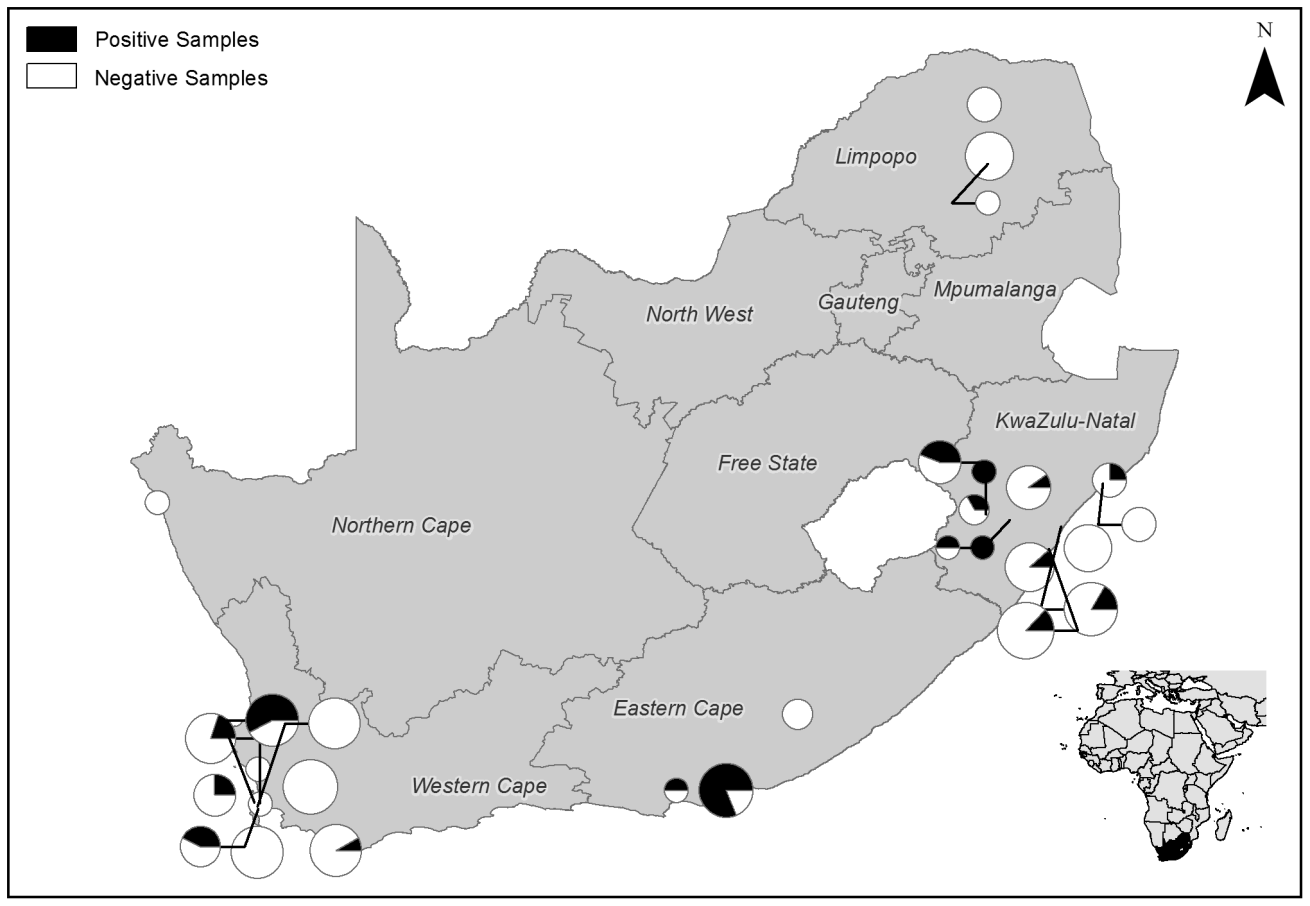
Map showing *Batrachochytrium dendrobatidis* swab sample sites for threatened species in South Africa. Pie-charts represent prevalence (black = positive samples, white = negative); size gives an indication of sample size.

In total, 392 swab samples were obtained from 17 (of 20) threatened South African frog species (Tables 1 and 3). Of the 17 threatened species tested, eight of these species were found to be positive for *Bd*, with an average prevalence of 14.8%. Prevalence in 23 non-threatened sympatric species from these sites was 24.4% (n = 127), which signified a significant difference in infection prevalence between threatened and non-threatened taxa (F[485] = 5.9456, p = 0.01515). Sampling was targeted at sites known to host threatened species and as such was not evenly distributed throughout the country. The majority of samples were therefore obtained in KwaZulu-Natal and the Western Cape lowlands (Figure 1). Of the 40 sites sampled, *Bd* was present at 20, with an overall infection prevalence of 17.5%. Prevalence varied considerably among taxa, from 79.4% in *Heleophryne hewitti* (n = 34) to 2.4% in *Xenopus gilli* (n = 41), with an average prevalence among threatened species of 14.5% (Table 3). The quantitative PCR indicated that the zoospore equivalents (indication of infection intensity) for positive samples varied widely and for statistical purposes values less than 0.05 were excluded, leaving 66 samples for analysis. Average zoospore equivalents ranged between 1375.6 for *Afrixalus spinifrons* (n = 8) and 0.16 for *Xenopus gilli* (n = 1). The original survey and infection data has been assimilated with the database of the Global *Bd* Mapping Project [6].

**Table 3:** *Batrachochytrium dendrobatidis* infection data from threatened (Red List 2010) South African frog species.

Species	Red List category	*N*	*Bd* Prevalence (%)	Mean zoospore equivalents
*Afrixalus spinifrons*	VU	20	62.5	1375.6
*Amietophrynus pantherinus*	EN	31	6.8	7.792
*Anhydrophryne rattrayi*	EN	3	0	**–**
*Breviceps gibbosus*	VU	2	0	–
*Breviceps macrops*	VU	4	0	–
*Breviceps sylvestris*	EN	21	0	–
*Capensibufo rosei*	VU	35	0	–
*Heleophryne hewitti*	EN	34	79.4	49.78
*Heleophryne rosei*	CR	34	50	18.97
*Hemisus guttatus*	VU	2	0	–
*Hyperolius horstockii*	VU	5	0	–
*Hyperolius pickersgilli*	CR	101	7	2.92
*Leptopelis xenodactylus*	EN	10	15.4	61.12
*Microbatrachella capensis*	CR	20	22.7	6.81
*Natalobatrachus bonebergi*	EN	19	0	–
*Vandijkophrynus amatolicus*	CR	1	0	–
*Xenopus gilli*	EN	50	2.4	0.16
**17 species**		**392 samples**	**Average: 14.8%**	**190.4**

### Predictive Modelling

The *Bd* occurrence data for South Africa included a total of 1, 577 samples that were tested for *Bd* either by PCR (n = 882) or histopathology (n = 695). Of these, 419 samples were positive for *Bd* infection, with an overall average prevalence of 26.6% (Table 4). The difference in infection prevalence between threatened (14.7% prevalence) and non-threatened (29.6% prevalence) species was also apparent at the national landscape scale (F[1575] = 13.529, p = 0.00024). The samples were obtained from both wild-caught and archived specimens, from across all nine provinces, and included approximately 62 species (25 genera), spanning occurrence dates between 1938 and 2012 (Table 5).

**Table 4:** Known *Batrachochytrium dendrobatidis* occurrence points in South Africa used for predictive distribution modelling (*Bd*+ =  infected).

Province	Tested individuals	Number of species (of which threatened)	*Bd*+ Samples	Geo-referenced *Bd*+ localities
EC	81	15 (5)	45	9
FS	133	7 (0)	26	14
GP	10	1 (0)	0	0
KZN	348	20 (5)	79	48
LP	219	8 (1)	12	7
MP	89	6 (0)	5	12
NC	137	10 (1)	108	3
NW	225	8 (0)	155	0
WC	616	15 (7)	200	28
**Total**	**1858**	**62** *	**630**	**121**

* each species was counted in every province that it occurred.

EC = Eastern Cape; FS = Free State; GP = Gauteng Province; KZN = KwaZulu-Natal; LP = Limpopo Province; MP = Mpumalanga Province; NC = Northern Cape; NW = North West Province; WC = Western Cape.

**Table 5:** *Batrachochytrium dendrobatidis* (*Bd*) occurrence records (1938–2012) from frog genera in South Africa used in the MaxEnt model.

Genus	N	Prevalence (%)	Geo-referenced
*Amietia*	466	38.8	39
*Afrixalus*	41	53.7	27
*Amietophrynus*	109	11.0	62
*Anhydrophryne*	4	0	2
*Arthroleptis*	4	0	0
*Breviceps*	32	0	32
*Cacosternum*	79	29.1	9
*Capensibufo*	32	0	32
*Chiromantis*	5	20	5
*Hadromophryne*	6	50	8
*Heleophryne*	85	63.5	70
*Hemisus*	7	42.8	3
*Hyperolius*	148	16.2	65
*Kassina*	31	19.4	4
*Leptopelis*	44	22.7	13
*Microbatrachella*	22	22.7	22
*Natalobatrachus*	13	0	13
*Phrynobatrachus*	24	37.5	5
*Ptychadena*	14	28.6	4
*Schismaderma*	23	60.9	9
*Semnodactylus*	4	0	0
*Strongylopus*	73	49.3	35
*Tomopterna*	46	28.3	10
*Vandijkophrynus*	2	50	2
*Xenopus*	263	9.9	212
**Total**	**1577**	**26.6%** **(Avg)**	**683** *

* Geo-referenced localities (GPS co-ordinates) include duplicates (multiple records from same locality).

**Figure 2:**
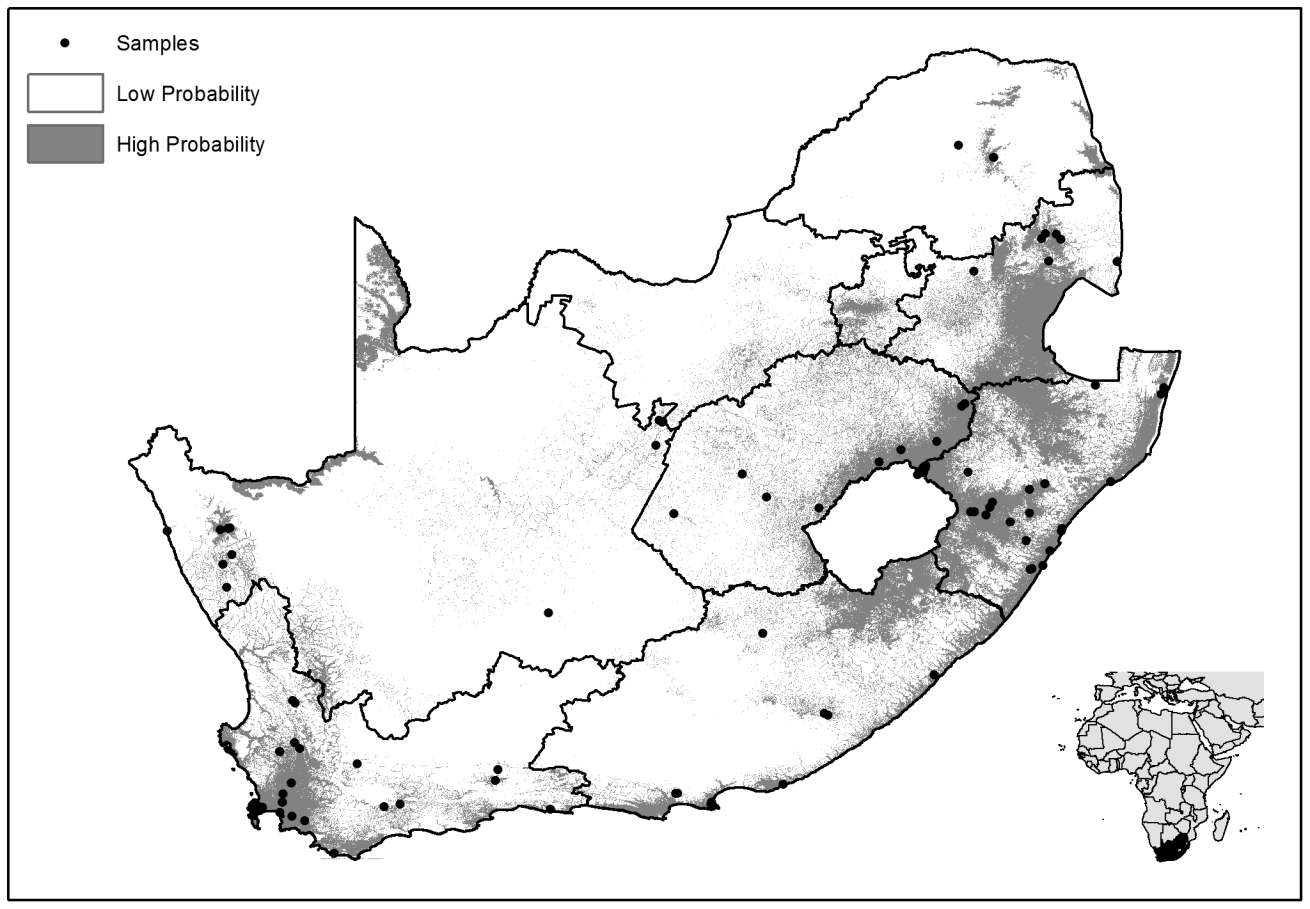
Probability threshold map for predicted occurrence of *Batrachochytrium dendrobatidis* in South Africa. Grey indicates areas of medium to high probability of occurrence at a 10% threshold.

Model accuracy was assessed by area under the curve (AUC) of the receiver operator characteristic (ROC), where 1 =  perfect prediction and 0.5 =  no better than random [54], [55]. For this model, mean test AUC for the *Bd* model was 0.885 indicating that the model provided a good fit to the data [56]. Analysis of variable contributions (Jack-knife tests) showed that “Precipitation of coldest quarter” (Bio 19), “Precipitation of Wettest Quarter” (Bio 16) and “Distance to water” had the highest predictive power when used in isolation. The resultant threshold map of the predictive model (Figure 2) indicates that occurrence of *Bd* infection is likely to be highest in the eastern and coastal regions of South Africa, with much of the drier central and northern inland regions unsuitable due to low precipitation and few water bodies that limit occupancy by amphibians and thus *Bd*. An exception is the Orange River basin in central South Africa that provides climatic refugia for *Bd* from the surrounding semi-arid environment. Our modeling results show that *Bd* is predicted to be particularly concentrated along the KwaZulu-Natal escarpment and highlands surrounding Lesotho as well as the lowlands of the Western Cape.

## Discussion

### *Batrachochytrium dendrobatidis* Occurrence

The significant role that *Bd* plays in amphibian declines has meant that understanding its prevalence and distribution is important for managing its spread and preventing introduction to suitable areas that remain disease-free [30], [57]. Knowledge of prevalence and distribution of *Bd* across South Africa has, until now, not been thoroughly assessed, in particular in terms of infection in Red List species. This study provides the first quantitative data on *Bd* infection prevalence in South Africa’s threatened species and makes use of *Bd* occurrence data from all known records in the country to model the predicted geographic distribution of infection.

Our analysis of the historic and present occurrence of *Bd* in South Africa shows that overall prevalence is 26.6% across all species and 14.5% for threatened species. The survey has identified taxonomic and geographic gaps in the South African *Bd* database, which can be used to direct future disease surveys. Approximately 39% of all South African species, and 85% of threatened South African species, have been screened for *Bd* infection to date, with the majority of sampling having been conducted in the Western Cape and KwaZulu-Natal provinces. Additional sampling is required in central South Africa, the Eastern Cape and Mpumalanga.

Prevalence of infection varied significantly among genera, for both threatened and non-threatened species. Of the nine threatened species for which no infection was detected, six were represented by samples fewer than six individuals. This number is below the minimum objective of 20 individuals required to yield statistically confident results [58], [59], [60] and therefore *Bd* status in these species could be a consequence of small sample size. Interestingly, none of the three threatened *Breviceps* species that were tested, including *B. sylvestris* (n = 28), were infected. This genus employs direct development, is strictly terrestrial, and has therefore little contact with aquatic environments that enhance disease transmission. Similarly, other terrestrial species showed either no (e.g. *Capensibufo*) or low infection intensity and prevalence (*Amietophrynus pantherinus*). In contrast, species with highly aquatic life-histories exhibited high infection prevalence. *Heleophryne hewitti* and *Afrixalus spinifrons* exhibit the highest prevalence of *Bd* infection (79.4, 50 and 62.5%, respectively) of all the threatened species. *Heleophryne* are riverine species that are closely associated with water [61] and as a consequence may be exposed to *Bd* transmission more frequently than terrestrial species.

*Afrixalus spinifrons* breeds in open water bodies (ponds, farm dams and wetlands) and males call from emergent vegetation at, or close to, the water’s surface pers. obs., [62]. This frequent contact with water may make this species more prone to infection [33]. The low prevalence and infection intensity in *X. gilli*, in spite of its aquatic existence, may be an indication of the resistance of this genus to the pathogen [16]. Compared with prevalence in non-threatened species (19.8%) at the same sites for which samples from Red List species were obtained, prevalence among threatened species was lower (14.5%), (range = 0–13 samples per site).

Neither clinical disease signs nor mortality were observed in any of the populations sampled. It should be noted however that three of the four Critically Endangered species tested positive for *Bd*, with the only exception being *V. amatolicus* for which only one specimen was screened, and which, again is largely a terrestrial species [63]. This places the Critically Endangered taxa in a particularly high risk category should pathogen virulence be exacerbated through sudden changes in environmental conditions [64].

It is important to recognise the distinction between infection with *Bd* that has no ill-effects, and infection that may have morbidity and mortality effects at the population level [65], [66], especially in species with high conservation priority. Whether the pathogen becomes lethal is subject to a complex array of factors, including life-history, immune defense system of host species, infection intensity and environmental context that influence the host-pathogen response [67]. Furthermore, host mortality is dependent on host age and size, duration of exposure to *Bd*, length of hibernation period and pathogen virulence [68]. Data from this study supports the concept that the presence of *Bd* does not necessarily cause declines in an endemic pathogen environment. The ease with which *Bd* infection can be detected with modern techniques has meant that investigations into disease-caused declines may have become somewhat neglected [69]. However in South Africa it does not appear that population declines have gone unnoticed due to the comprehensive database that comprises more than a decade of survey data, in addition to archived records spanning over seven decades.

### Predicted Distribution

Predictive distribution models provide a practical solution for minimising the rate of false negatives at the population scale and for optimising sample collection [30]. Distribution of *Bd* is often not homogenous even in regions in which the pathogen is widespread and *Bd* is able to tolerate a broad range of climatic variables across varying altitudes [30], [31]. Until now, a fine scale model of predicted distribution for *Bd* in South Africa has not been available. Our MaxEnt model generated from up-to-date data shows patterns consistent with models from other regions of the world, e.g. [30], in that *Bd* distribution is correlated with generally cooler and wetter areas and excluded from arid areas. The areas with the highest predicted distribution of *Bd* coincide with the areas that host the highest frog diversity and endemicity in South Africa (KwaZulu-Natal and the Western Cape) [12]. However, these regions are also those with the highest number of samples and this may have influenced the model. The model also indicated that *Bd* can occur in a wide range of locations with conditions ranging from the warm and wet lowlands of KwaZulu-Natal to the more climatic extremes of the Drakensberg escarpment. The central and north-western regions of South Africa were predicted to be least suitable for *Bd*. More records are needed from the relatively wet Orange River basin that dissects this otherwise dry region to test the extent to which this corridor is able to expand the distribution of *Bd* from the more suitable east of the country to the less suitable western extreme.

This model may underestimate the full extent of *Bd* distribution given that zoospores of *Bd* are able to survive in the absence of an amphibian host for up to seven weeks in water or saprophytically on algae or exoskeleton material [70] and for up to 3 months in sterile sand or on bird feathers [71]. A mathematical model developed by [34] showed that the longer *Bd* could persist in water, the more likely it was to cause local extinction of an experimentally infected host (*Bufo bufo*). The capacity to persist for long periods may be partly responsible for the pathogen’s ability to drive amphibian declines. It has recently become known that *Bd* is not only able to infect crayfish, but is able to transmit from the alternative host to amphibians [72]. This suggests that the potential distribution of *Bd* is not only governed by amphibian host distribution and climate envelope, but at least to some extent it is also determined by the distribution of possible alternative hosts. No evidence for *Bd* infecting alternative hosts is available in South Africa, inasmuch it has not been investigated.

The model indicated that the distribution of *Bd* is most strongly influenced by precipitation of the wettest season and distance from water. This is expected given *Bd*’s low tolerance for desiccation [73], [74] and dependence on the presence of permanent water for the transmission of aquatic zoospores [75]. The variables “Mean temperature of coldest quarter (°C)” (11.8%) and “Topography” (19.6%) also contributed significantly to the model, indicating *Bd*’s tolerance for cool temperatures between 10 and 23°C [1], [76], which overlaps with the range of mean winter temperatures for South Africa (−3 to 15°C) and coincides with temperatures in valleys within the Orange River catchment. The influence of topography can be explained further by *Bd*’s habitat requirements of low-lying water-bodies and generally cooler temperatures that often persist in valleys, especially when vegetation cover is sufficient to allow a cooler environment.

Without exception, all of South Africa’s threatened species have distribution ranges that overlap with the predicted distribution for *Bd* and as such are thus potentially exposed to *Bd* infection. As has been shown by the results of the prevalence of *Bd* infection in threatened species, those with more aquatic life-histories and that are associated with permanent water bodies are most susceptible to infection and risk of disease [32]. Discounting *Bd* as a threat to terrestrial and direct breeding threatened species should also not be taken for granted considering that some species could be susceptible to infection and that there is partial overlap in distribution with predicted *Bd* range. Combining life-history factors with knowledge of pathogen distribution will benefit disease risk analysis [27], [31].

### Conservation Recommendations

Our model provides a clearer indication of where *Bd* infection is likely to occur within South Africa, and can be used for identifying species that are at risk of *Bd*-related declines. [27] identify high-altitude, range-restricted, aquatic species with low fecundity as being those most susceptible to rapid declines. Cognisance of this, together with knowledge of pathogen distribution can help identify species that may be at risk of experiencing *Bd*-related declines. The threatened South African anurans that fall under these criteria, and which exhibit high infection intensity, may be particularly susceptible to outbreaks of chytridiomycosis. Another species, although not in the highest threat categories, that warrants attention is *Amietia vertebralis* (Near Threatened) which is endemic to the north-western Drakensberg of KwaZulu-Natal and Lesotho. Although declines have not yet been detected, populations of *A. vertebralis* are the only southern African species for which intermittent mortality has been observed. The effects of external stressors, such as climate change, may exacerbate the impact of *Bd* on this high altitude species.

Because of the variable and often unpredictable outcome of *Bd* infection to host fitness, management of this disease should be context specific. Various management strategies have been suggested including mitigating disease at population level and minimising disease threat by regulating amphibian trade [58], [77]. The Office International des Epizooties (OIE) has developed standards pertaining to trade of amphibians and *Bd* infection [78]. However the export of wild-caught *Xenopus laevis* from South Africa is ongoing, without screening for *Bd* being conducted, despite this being identified as a major means of initial dissemination of the pathogen [17], [79]. This undoubtedly poses a risk to native amphibians for importing countries and necessitates the development and implementation of stringent regulations for the international trade of amphibians.

A vigilant approach should also be employed to prevent the introduction of a novel strain that could be potentially lethal to certain South African species. Although *Bd* appears to now be endemic to many regions of the world, with populations able to persist with low levels of infection [5], [80], experimentation has shown that infection with novel strains of *Bd* can cause death even when local strains do not [81]. Coupled with the potential unknown effect of additional stressors such as climate change and anthropomorphic habitat transformation, South Africa could still experience population declines as a result of chytridiomycosis. As such, identification of potentially susceptible species and likely areas of infection are essential first steps for any form of mitigation from the development of a surveillance program to *ex-situ* population management.

## References

[1] Longcore JE, Pessier AP, Nichols DK (1999) *Batrachochytium dendrobatidis* gen. Et sp. Nov., a chytrid pathogenic to amphibians. Mycologia 91: 219–227.

[2] Daszak P, Cunningham AA, Hyatt AD, Green DE, Speare R (1999) Emerging infectious diseases of wildlife–threats to biodiversity and human health. Emerg Infect Dis 5: 735–748.10603206

[3] Skerratt LF, Berger l, Speare R, Cashins S, Macdonald KR, et al (2007) Spread of chytridiomycosis has caused the rapid global decline and extinction of frogs. Ecohealth 4: 125–134.

[4] Fisher MC, Garner TWJ, Walker SF (2009) Global emergence of *Batrachochytrium dendrobatidis* and amphibian chytridiomycosis in space, time, and host. Annu Rev Microbiol 63: 291–310. 19575560 10.1146/annurev.micro.091208.073435

[5] Briggs CJ, Knapp RA, Vrendenburg VT (2010) Enzootic and epizootic dynamics of the chytrid fungal pathogen of amphibians. Proc Natl Acad Sci USA 1–6: doi: 10.1073/pnas.0912886107. 10.1073/pnas.0912886107 PMC2906864 20457916

[6] Aanensen DM (2012) Bd-maps. Available: http://www.bd-maps.net. Accessed 2012 April 22.

[7] Daszak P, Lips K, Alford R, Carey C, Collins JP, et al.. (2007) Chapter 4: Infectious Diseases. In Gascon, C, Collins, JP, Moore RD, Church DR, McKay J. et al. editors. The Amphibian Conservation Action Plan. IUCN.

[8] Hyatt AD, Boyle DG, Olsen V, Boyle DB, Berger L, et al (2007) Diagnostic assays and sampling protocols for the detection of *Batrachochytrium dendrobatidis* . Dis Aquat Organ 73: 175–192. 17330737 10.3354/dao073175

[9] Daszak P, Cunningham AA, Hyatt AD (2003) Infectious disease and amphibian population declines. Divers Distrib 9: 141–150. 10.3201/eid0506.990601 PMC2640803 10603206

[10] Burrowes PA, Joglar RL, Green DE (2004) Potential causes for amphibian declines in Puerto Rico. Herpetologica 60 (2): 141–154.

[11] IUCN (2012) IUCN Red List of threatened species. Version 2012.2. Available: http://www.iucnredlist.org. Accessed 2012 Dec 04.

[12] Measey GJ (2011) Ensuring a future for South Africa’s frogs: a strategy for conservation research. SANBI Biodiversity Series 19. South African National Biodiversity Institute, Pretoria.

[13] South African Frog Re-Assessment Group (SA-FRoG) & IUCN SSC Amphibian Specialist Group (2010) In:IUCN 2011. IUCN Red List of Threatened Species. Version 3.1 Available: http://www.iucnredlist.org/apps/redlist/details/10644/0. Accessed 2011 December 2.

[14] Goodman P (2000) Determining the conservation value of land in KwaZulu–Natal. Final report. Biodiversity Division, KwaZulu–Natal Nature Conservation Service, Pietermaritzburg.

[15] Mittermeier RA, Gil PR, Hoffman M, Pilgrim J, Brooks T, et al.. (2005) Hotspots revisited: earth's biologically richest and most endangered ecoregions. The university of chicago press.

[16] Weldon C, Du Preez LH, Hyatt AD, Muller R, Speare R (2004) Origin of the amphibian chytrid fungus. Emerg Infect Dis 10(12): 2100–2105. 15663845 10.3201/eid1012.030804 PMC3323396

[17] Weldon C, De Villiers AL, Du Preez LH (2007) Quantification of the trade in *Xenopus laevis* from South Africa, with implications for biodiversity conservation. Afr J Herpetol 56(1): 77–83.

[18] Soto-Azat C, Clarke BT, Poynton JC, Cunningham AA (2009) Widespread historical presence of *Batrachochytrium dendrobatidis* in African pipid frogs. Divers Distrib 16: 126–131 doi: –10.1111/j.1472–4642.2009.00618.x

[19] Goldberg TL, Readel AM, Lee MH (2007) Chytrid Fungus in Frogs from an Equatorial African Montane Forest in western Uganda. J Wildl Dis 43: 521–524. 17699093 10.7589/0090-3558-43.3.521

[20] Greenbaum E, Kusamba C, Aristote MM, Reed K (2008) Amphibian chytrid fungus infections in *Hyperolius* (Anura: Hyperoliidae) from eastern Democratic Republic of Congo. Herpetol Rev 39: 70–73.

[21] Kielgast J, Rödder D, Veith M, Lötters S (2010) Widespread occurrence of the amphibian chytrid fungus in Kenya. Anim Conserv 13: 1–8.

[22] Bell RC, Garcia AVG, Stuart BL, Zamudio KR (2011) High prevalence of the amphibian chytrid pathogen in Gabon. EcoHealth 8: 116–120. 21210295 10.1007/s10393-010-0364-4

[23] Doherty-Bone TM, Gonwouo NL, Ohst T, Weldon C, Perkins M, et al.. (2012) *Batrachochytrium dendrobatidis* in amphibians of Cameroon, including the first records of infected caecilian hosts. Dis Aquat Organ: In Press. 10.3354/dao02557 23446968

[24] Weldon C, Du Preez LH (2004) Decline of the Kihansi Spray Toad, *Nectophrynoides asperginis*, from the Udzungwa Mountains, Tanzania. Froglog 62: 2–3.

[25] Penner J, Adum GB, McElroy MT, Doherty-Bone T, Hirschfeld M, et al (2013) West Africa - A Safe Haven for Frogs? A Sub-Continental Assessment of the Chytrid Fungus (*Batrachochytrium dendrobatidis*). PLoS ONE 8(2): e56236 doi:10.1371/journal.pone.0056236 23426141 10.1371/journal.pone.0056236 PMC3572032

[26] Minter LR, Burger M, Harrison JA, Braack HH, Bishop PJ, et al.. (2004) Atlas and Red Data Book of the Frogs of South Africa, Lesotho and Swaziland. SI/MAB Series #9. Smithsonian Institution, Washington, DC, 360 p.

[27] Bielby J, Cooper N, Cunningham AA, Garner TWJ, Purvis A (2008) Predicting susceptibility to future declines in the world’s frogs. Conserv Lett 1: 82–90.

[28] Rödder D, Kielgast J, Bielby J, Schmidtlein S, Bosch J, et al (2009) Global amphibian extinction risk assessment for the panzootic chytrid fungus. Diversity 1: 52–66.

[29] Ron SR (2005) Predicting the distribution of the amphibian pathogen *Batrachochytrium dendrobatidis* in the new world. Biotropica 37(2): 209–221.

[30] Adams MJ, Chalegren ND, Reinitz D, Cole RA, Rachowicz LJ, et al (2010) Using occupancy models to understand the distribution of an amphibian pathogen, *Batrachochytrium dendrobatidis* . Ecol Appl 20(1): 289–302. 20349848 10.1890/08-2319.1

[31] Murray KA, Retallick RWR, Puschendorf R, Skerratt L, Rosauer D, et al (2011) Assessing the spatial patterns of disease risk to biodiversity: implications for the management of the amphibian pathogen, *Batrachochytrium dendrobatidis* . J Appl Ecol 48: 163–173.

[32] Berger L, Speare R, Hines HB, Marantelli G, Hyatt AD, et al (2004) Effect of season and temperature on mortality in amphibians due to chytridiomycosis. Aust Vet J 82 (7): 434–439. 10.1111/j.1751-0813.2004.tb11137.x 15354853

[33] Johnson ML, Speare R (2003) Survival of *Batrachochytrium dendrobatidis* in water: quarantine and disease control implications. Emerg Infect Dis 8: 922–925. 10.3201/eid0908.030145 PMC3020615 12967488

[34] Mitchell KM, Churcher TS, Garner TWJ, Fisher MC (2007) Persistence of the emerging pathogen *Batrachochytrium dendrobatidis* outside the amphibian host greatly increases the probability of host extinction. Proc Biol Sci. doi:10.1098/rspb.2007.1356 10.1098/rspb.2007.1356 PMC2593721 18048287

[35] Brem F, Mendelson JR III, Lips KR (2007) Field–sampling protocol for *Batrachochytrium dendrobatidis* from living amphibians, using alcohol–preserved swabs. Version 1.0 (18 July 2007). Available: http://www.amphibianark.org/pdf/Field%20sampling%20protocol%20for%20amphibian%20chytrid%20fungi%201.0.pdf. Accessed 2013 Jun 18.

[36] St-Hilaire S, Tatarian T, Prasad A, Peeler E, Thrush M (2007) A tool for estimating the risk of anthropogenic spread of *Batrachochytrium dendrobatidis* (Bd) between water bodies. EcoHealth doi: 10.1007/s10393–009–0227-z. 10.1007/s10393-009-0227-z 19421816

[37] Boyle DG, Boyle DB, Olsen V, Morgan JAT, Hyatt AD (2004) Rapid quantitative detection of chytridiomycosis (*Batrachochytrium dendrobatidis*) in amphibian samples using real–time Taqman PCR assay. Dis Aquat Organ 60: 141–148. 15460858 10.3354/dao060141

[38] Annis SL, Dastoor FP, Ziel H, Daszak P, Longcore JE (2004) A DNA-based assay identifies *Batrachochytrium dendrobatidis* in amphibians. J Wildl Dis 40(3): 420–428. 15465708 10.7589/0090-3558-40.3.420

[39] Phillips SJ, Anderson RP, Schapire RE (2006) Maximum entropy modelling of species geographic distributions. Ecol Modell 190: 231–259.

[40] Elith J, Phillips SJ, Hastie T, Dudík M, En Chee Y, et al (2011) A statistical explanation of Maxent for ecologists. Divers Distrib 17: 43–57.

[41] Jackson CR, Robertson MP (2011) Predicting the potential distribution of an endangered cryptic subterranean mammal from few occurrence records. J Nat Conserv 19: 87–94.

[42] Stabach JA, Laporte N, Olupot W (2009) Modeling habitat suitability for Grey Crowned-cranes (*Balearica regulorum gibbericeps*) throughout Uganda. Int J Biodivers Conserv 1: 177–186.

[43] Phillips SJ, Dudík M, Schapire RE (2004) A maximum entropy approach to species distribution modelling. 21^st^ International conference on machine learning, Banff, Canada.

[44] Gibson L, Barrett B, Burbidge A (2007) Dealing with uncertain absences in habitat modelling: A case study of a rare ground-dwelling parrot. Divers Distrib 13: 704–713.

[45] Pearson RG, Raxworthy CJ, Nakamura M, Peterson TA (2007) Predicting species distributions from for small numbers of occurrence records: A test case using cryptic geckos in Madagascar. J Biogeogr 34: 102–117.

[46] Hernandez P, Franke I, Herzog S, Pacheco V, Paniagua L, et al (2008) Predicting species distributions in poorly-studied landscapes. Biodivers Conserv. 17: 1353–1366.

[47] Ward D (2007) Modelling the potential geographic distribution of invasive ant species in New Zealand. Biol Invasions 9: 723–735.

[48] Tinoco BA, Astudillon PX, Latta SC, Graham CH (2009) Distribution, ecology and conservation of an endangered Andean hummingbird: The violet-throated metaltail (*Metallura baroni*). Bird Conserv Int 19: 63–76.

[49] Hijmans RJ, Cameron SE, Parra JL, Jones PG, Jarvis A (2005) Very high resolution interpolated climate surfaces for global land areas. Int J Climatol 25: 1965–1978.

[50] Mucina L, Rutherford MC, Powrie LW (2006) Vegetation Map of South Africa, Lesotho and Swaziland, 1:1 000 000 Scale Sheet Maps. Pretoria: SANBI.

[51] ESRI (2011) ArcGIS desktop: Release 10. Redlands, ca: Environmental Systems Research Institute.

[52] Phillips SJ, Dudík M, Elith J, Graham CH, Lehmann A, et al (2009) Sample selection bias and presence-only distribution models: implications for background and pseudo-absence data. Ecol Appl 19(1): 181–197. 19323182 10.1890/07-2153.1

[53] Fielding AH, Bell JF (1997) A review of methods for the assessment of prediction errors in conservation presence/absence models. Environ Conserv 24(1): 38–49.

[54] Phillips SJ, Dudík M (2008) Modeling of species distributions with Maxent: New extensions and a comprehensive evaluation. Ecography 31: 161–175.

[55] Wiley EO, McNyset KM, Peterson AT, Robins CR, Stewart AM (2003) Niche modelling and geographic range predictions in the marine environment using a machine-learning algorithm. Oceanography 16(3): 120–127.

[56] Wisz MS, Hijmans RJ, Li J, Peterson AT, Graham CH, et al (2008) Effects of sample size on the performance of species distribution models. Divers Distrib 14: 763–773.

[57] Woodhams DC, Bosch J, Briggs CJ, Cashins S, Davis lR, et al (2011) Mitigating amphibian disease: strategies to maintain wild populations and control chytridiomycosis. Front Zool 8 (8): 1–23. 10.1186/1742-9994-8-8 PMC3098159 21496358

[58] Digiacomo RF, Koepsell TD (1986) Sampling for detection of infection or disease in animal populations. J Am Vet Med Assoc 189: 22–23. 3733495

[59] Skerratt LF, Berger L, Hines HB, McDonald KR, Mendez D, et al (2008) Survey protocol for detecting chytridiomycosis in all Australian frog populations. Dis Aquat Org 80: 85–94. 18717061 10.3354/dao01923

[60] Skerratt LF, McDonald KR, Mendez D, Berger L, Hines HB, et al (2010) Application of the survey protocol for chytridiomycosis to Queensland, Australia. Dis Aquat Org. Special. Chytridiomycosis: an emerging disease 92: 117–129. 10.3354/dao02272 21268974

[61] Du Preez LH, Carruthers V (2009) A complete guide to the frogs of southern Africa. Struik Nature, Cape Town, 200–212.

[62] Pickersgill M, Burger M, Bishop PJ (2004) *Afrixalus spinifrons* species account. In: Minter LR, Burger M, Harrison JA, Braack HH, Bishop PJ, et al.. editors. Atlas and Red Data book of the frogs of South Africa, Lesotho and Swaziland. Si/mab series #9. Smithsonian Institution, Washington, DC. 131–133.

[63] Boycott RC (2004) *Bufo amatolicus* species account. In: Minter LR, Burger M, Harrison JA, Braack HH, Bishop PJ, et al.. editors. Atlas and Red Data book of the frogs of South Africa, Lesotho and Swaziland. Si/mab series #9. Smithsonian Institution, Washington, DC. 56–58.

[64] Andre SE, Parker J, Briggs CJ (2008) Effect of temperature on host response to *Batrachochytrium dendrobatidis* infection in the mountain yellow-legged frog (*Rana muscosa*). J Wildl Dis 44: 716–720. 18689660 10.7589/0090-3558-44.3.716

[65] Forzán MJ, Vanderstichel R, Hogan NS, Teather, Wood J (2010) Prevalence of *Batrachochytrium dendrobatidis* in three species of wild frogs on Prince Edward Island, Canada. Dis Aquat Organ 91: 91–96. 21387987 10.3354/dao02244

[66] Voyles J, Young S, Berger L, Campbell C, Voyles WF, et al (2009) Pathogenesis of chytridiomycosis, a cause of catastrophic amphibian declines. Science 326: 582–585. 19900897 10.1126/science.1176765

[67] Longo AV, Burrowes PA (2010) Persistence with chytridiomycosis does not assure survival of direct-developing frogs. EcoHealth 7: 185–195. 20585971 10.1007/s10393-010-0327-9

[68] Garner TWJ (2007) Experimental evidence of innate immunity: a matter of design, convenience or constraints? Anim Conserv 10: 418–419.

[69] Duffus ALJ (2009) Chytrid blinders: What other disease risks to amphibians are we missing? EcoHealth 6: 335–339. 20135193 10.1007/s10393-009-0271-8

[70] Johnson ML, Speare R (2003) Survival of *Batrachochytrium dendrobatidis* in water: Quarantine and disease control implications. Emerg Infect Dis 8: 922–925. 10.3201/eid0908.030145 PMC3020615 12967488

[71] Johnson ML, Speare R (2005) Possible modes of dissemination of the amphibian chytrid *Batrachochytrium dendrobatidis* in the environment. Dis Aquat Organ 65: 181–186. 16119886 10.3354/dao065181

[72] McMahon TA, Brannelly LA, Chatfield MWH, Johnson PTJ, Joseph MB, et al (2012) Chytrid fungus *Batrachochytrium dendrobatidis* has non-amphibian hosts and releases chemicals that cause pathology in the absence of infection. Proc Natl Acad Sci USA 110 210–215. 23248288 10.1073/pnas.1200592110 PMC3538220

[73] Berger L, Hyatt AD, Speare R, Longcore JE (2005) Life cycle stages of the amphibian chytrid *Batrachochytrium dendrobatidis* . Dis Aquat Organ 68: 51–63. 16465834 10.3354/dao068051

[74] Johnson ML, Berger L, Phillips L, Speare R (2003) Fungicidal effects of chemical disinfectants, UV light, desiccation and heat on the amphibian chytrid, *Batrachochytrium dendrobatidis* . Dis Aquat Organ 57: 255–260. 14960039 10.3354/dao057255

[75] Berger L, Speare R, Daszak P, Green DE, Cunningham AA, et al (1998) Chytridiomycosis causes amphibian mortality associated with population declines in the rain forests of Australia and Central America. Proc Natl Acad Sci USA 95: 9031–9036. 9671799 10.1073/pnas.95.15.9031 PMC21197

[76] Piotrowski JS, Annis SL, Longcore JE (2004) Physiology of *Batrachochytrium dendrobatidis*, a chytrid pathogen of amphibians. Mycologia 96(1): 9–15. 21148822

[77] Kriger KM, Hero J-M (2009) Chytridiomycosis, amphibian extinctions, and lessons for the prevention of future panzootics. Ecohealth 6(1): 148–151. 19421815 10.1007/s10393-009-0228-y

[78] OIE Website. World Organisation for Animal Health. Chapter 8.1. Infection with *Batrachochytrium dendrobatidis* Available: www.oie.int/fileadmin/Home/eng/Health_standards/aahc/2010/en_chapitre_1.8.1.htm. Accessed 2013 Jun 18.

[79] Weldon C, Fisher MC (2011) The effect of trade–mediated spread of amphibian chytrid on amphibian conservation. In: IOM. Fungal diseases: an emerging challenge to human, animal, and plant health. Washington DC: The National Academies Press. 22259817

[80] Tobler U, Schmidt BR (2010) Within- and among-population variation in chytridiomycosis–induced mortality in the toad *Alytes obstetricians*. PLoS ONE 5 (6) e10927. 10.1371/journal.pone.0010927 PMC2880007 20532196

[81] Gahl MK, Longcore JE, Houlahan JE (2011) Varying responses of northeastern north American amphibians to the chytrid pathogen *Batrachochytrium dendrobatidis* . Conserv Biol 26 (1): 135–141. 10.1111/j.1523-1739.2011.01801.x 22181933

